# Plasma untargeted metabolomics with proteinase K discloses phospholipid signature associated with pulmonary arterial hypertension

**DOI:** 10.1038/s41598-023-42293-w

**Published:** 2023-09-15

**Authors:** Renata Wawrzyniak, Margot Biesemans, Alicja Kugacka-Dąbrowska, Ewa Lewicka, Rafał Bartoszewski, Michał J. Markuszewski

**Affiliations:** 1 Department of Biopharmaceutics and Pharmacodynamics, Medical University of Gdańsk, Hallera 107, 80-416 Gdańsk, Poland; 2 Department of Cardiology and Electrotherapy, Medical University of Gdansk, Debinki 7, 80-210 Gdańsk, Poland; 3 Department of Biophysics, Faculty of Biotechnology, University of Wroclaw, Ul. F. Joliot-Curie 14A, 50-383 Wrocław, Poland

**Keywords:** Metabolomics, Phospholipids

## Abstract

Pulmonary arterial hypertension is a rare but life-threatening and clinically heterogeneous disease. The diagnostic schedule of this disorder is complex, and no specific indicator of the arterial etiology has been explored. In this study, untargeted plasma metabolomics was applied to evaluate the metabolic fingerprints of pulmonary arterial hypertension patients. Plasma samples were prepared using a new approach, which applies proteinase K during the sample preparation procedure to increase the metabolite coverage. The metabolic fingerprints were determined via LC–MS and subsequently analyzed with the use of both uni- and multivariate statistics. A total of 21 metabolites were discovered to be significantly altered in pulmonary arterial hypertensive patients. The metabolites were mainly related to the phospholipid metabolic pathways. In this study, decreases were found in the phosphatidylcholines (PCs) [PC(32:0), PC(40:7), PC(42:7)], phosphatidylethanolamine PE(18:0/18:2), lysophosphatidylethanolamines (LPEs) [LPE(22:6), LPE(18:2), LPE(18:0), LPE(20:4), LPE(20:1), LPE(20:0)], lysophosphatidylcholine LPC(20:4) and lysophosphatidylserine LPS(19:0), as well as increase of sphingomyelin SM(36:2), in the plasma samples of pulmonary arterial hypertensive patients in comparison to the control group. Besides their function as components of the biological membranes, these metabolites are also involved in the intracellular signaling pathways that are related to cell proliferation and apoptosis. The results obtained during this study confirm the potential of (untargeted) metabolomics to identify the molecular characteristics of the pathophysiology of pulmonary arterial hypertension. The clinical relevance of this study constitutes the selection of a metabolic panel that can potentially detect and properly diagnose the disease.

## Introduction

Pulmonary hypertension (PH) is a rare but life-threatening and clinically heterogenous disease, characterized by vascular remodeling and progressive elevation in pulmonary vascular resistance (PVR)^1^. Regarding the WHO, ESC (European Society of Cardiology) 2016, and the Sixth World Symposium on Pulmonary Hypertension guidelines, PH is classified into five groups based on the clinical symptoms, pathological hallmarks, hemodynamic parameters, and treatment strategy^2,3^.

Pulmonary arterial hypertension (PAH) constitutes a term that specifically indicates WHO group 1 and includes three main subgroups, namely: heritable PAH (HPAH), drug-induced PAH and idiopathic PAH (IPAH). The diagnostic procedure of PAH is complex, as other types of PH have to be excluded, and no specific indicator of the arterial etiology has been discovered. Right heart catheterization (RHC) still remains the gold standard for a proper PAH diagnosis and the key guidance of the treatment. Therefore, there is still a need to explore noninvasive and more specific diagnostic markers of PAH. Metabolomics is a research discipline that focuses on the analysis of small molecules in biological samples^4^. The main aim of metabolomics is to gain insight into the metabolome changes resulting from pathophysiological mechanisms. Pathophysiological changes on every biological level will eventually lead to changes in the concentrations of metabolites.

The holistic approach of metabolomics, especially an untargeted analysis, has the advantage of considering as many metabolites as possible, as opposed to biomarker tests that test only a small number of metabolites. However, disease pathophysiology on the metabolome level can be very complex, and it might not be entirely grasped by testing only a few disease biomarkers. This is why metabolomics has been applied to study several disease mechanisms to gain an understanding of the pathophysiology on a metabolic level. In addition, new diagnostic tests can be developed based only on the analysis of a broad metabolome coverage. A growing body of evidence shows that pathological hallmarks of PAH include mitochondrial dysfunction, interruption of glycolysis, increased fatty acid metabolism and changes in oxidation pathways^5,6^. However, the majority of the current knowledge regarding the pathogenesis of the disease has been derived from animal-based studies^7–10^.

Additionally, most of the recent metabolomics-based studies of PH in the human population have considered different etiologies of the disease^11,12^ or were focused on the analysis of lung tissue samples^13,14^. Evaluation of the tissue metabolic signature provides a specific insight into the molecular mechanisms of PAH, however, it is invasive and limited by the number and amount of samples available for analysis. Blood plasma constitutes a less invasive and readily available biofluid, but it contains a high amount of proteins, which are best to be removed from the matrix before the analytical measurements on MS-based instruments. The conventional plasma preparation procedures applied for untargeted metabolomics remove plasma proteins by precipitation with an organic solvent. The major disadvantage of the conventional plasma preparation procedure is the loss of all metabolites that are primarily retrieved at the surface—or interior of proteins. Extensive interactions through numerous interaction mechanisms have already been proven between small molecules and proteins^15^. Accordingly, metabolites that perform their biological function at the protein level (for example, a regulator in a gene transcription complex) might be lost using the conventional plasma preparation procedure. Yet, this specific subset of molecules may be even more interesting to investigate for the mapping of disease-related metabolites. Therefore, in this study, untargeted plasma metabolomics was applied to evaluate the metabolic fingerprints of a clinically homogenous group of PAH. Moreover, a new plasma preparation procedure was used by applying proteinase K to enrich the metabolome coverage via proteolytic degradation of the plasma proteins. This approach ensures the relaxation of native proteins’ tertiary structure and the metabolites’ release from the protein interactions. To the best of the authors' knowledge, it has been applied for the first time in a clinically relevant set of PAH samples, which could provide a better insight into the metabolite changes involved in the disease pathogenesis.

## Materials and methods

### Study groups

This observational study included 43 adult PAH patients treated according to the Polish Health Fund program in the Department of Cardiology of the Medical University of Gdansk. The study was performed in accordance with the Declaration of Helsinki and was approved by the Ethical Committee of the Medical University of Gdansk (number of approval: NKBBN/204/2018). The diagnosis of PAH was established via RHC, with a mean pulmonary arterial pressure ≥ 25 mmHg at rest, a pulmonary artery wedge pressure ≤ 15 mmHg and in the absence of any other causes of precapillary pulmonary hypertension. Amongst the PAH patients, approximately 50% were characterized as the idiopathic subtype. Therefore, patients with PH due to left heart disease, lung disease, thromboembolic, or other rare disorders were excluded. A routine evaluation was performed every 6 months and included PAH medical history and co-morbidities, assessment of the WHO functional class, physical examination, electrocardiography (ECG), echocardiography, blood sample analysis, and non-encouraged 6-min walk test (6MWT), performed according to American Thoracic Society recommendations. The baseline demographic and clinical characteristics of PAH patients enrolled in this study are provided in Table 1. The control group (n = 36) consisted of age (p = 0.506), sex (p = 0.193), and body-surface-area (p = 0.114) matched volunteers drawn from a general population. The age and body-surface-area for the control group were: 44.0 ± 16.4 (average years ± standard deviation) and 23.3 ± 2.8 (average kg/m^2^ ± standard deviation), respectively. The control group included 23 females. All participants included in this study signed their informed consent forms.

**Table 1:** Baseline clinical characteristics of patients with PAH.

Variables			
Age (years)	46 ± 18		
Females (n/%)	27 (64)		
Height (cm)	162 ± 10		
Weight (kg)	67 ± 15		
BMI (kg/m^2^)	26 ± 6		
PAH classification (n/%)			
Idiopathic	22 (51)		
Connective tissue disease	3 (7)		
Congenital heart disease	18 (42)		
WHO functional class (n/%):			
I	1 (2)		
II	18 (42)		
III	16 (37)		
IV	8 (19)		
Co-morbidities (n/%)			
Arterial hypertension	10 (23)		
Hypothyreosis	12 (28)		
Diabetes mellitus	5 (12)		
Renal failure	4 (9)		
Coronary artery disease	6 (14)		
Lung disease	8 (19)		
Paroxysmal/persistent AF	2 (5)		

	LQ	Median	UQ
Physiological measurements			
HR (beats/min)	68.50	77.0	85.50
SBP (mmHg)	101.75	115.50	125.75
DBP (mmHg)	65.25	70.00	77.00
Pulse pressure (mmHg)	34.00	41.50	52.50
Laboratory measurements			
BNP (pg/mL)	22.0	51.0	110.00
Hemoglobin (g/dL)	13.33	16.20	19.17
PLT (thou./uL)	153.00	187.50	226.50
Sodium (mmol/L)	136.25	138.00	140.00
Iron (µg/dL)	55.00	79.00	103.00
Uric acid (mg/dL)	5.40	6.40	7.80
Bilirubin (mg/dL)	0.60	1.08	1.44
GGT (U/l)	16.0	29.00	56.50
ALP (U/l)	59.0	72.0	89.0
AST (U/l)	15.0	19.0	24.0
ALT (U/l)	13.0	18.0	25.0
Creatinine (mg/dL)	0.72	0.85	1.09
6MWT (m)	329.50	399.0	500.50
Right heart catheterization parameters			
mPAP (mmHg)	46.0	56.0	71.0
PCWP (mmHg)	6.25	9.0	11.0
mRAP (mmHg)	3.75	5.50	8.0
CI (ml/kg/min)	1.99	2.60	3.05
PVR (Wood units)	6.55	10.84	15.55

*HR* heart rate, *SBP* systolic blood pressure, *DBP* diastolic blood pressure, *BNP* brain natriuretic peptide, *PLT* platelet count, *GGT* gamma-glutamyltranspeptidase, *ALP* alkaline phosphatase, *AST* aspartate aminotransferase, *ALT* alanine aminotransferase, *6MWT* 6-min walk test, *mPAP* mean pulmonary artery pressure, *PCWP* pulmonary capillary wedge pressure, *mRAP* mean right atrial pressure, *CI* cardiac index, *PVR* pulmonary vascular resistance, *n* number of patients, data presented as mean ± standard deviation for data that has a normal distribution or as median and lower quartile (LQ) as well as upper quartile (UQ) for data that does not have a normal distribution.

### Sample collection

Blood samples were collected at the Department of Cardiology & Electrotherapy of the 2nd Department of Cardiology in the University Clinical Centre of the Medical University of Gdańsk. The blood samples were collected according to the guidelines of the independent ethics committee of the Medical University of Gdańsk (number of approval: NKBB/204/2018) and in accordance with the Declaration of Helsinki. All patients and controls declared their informed consent. Fasting peripheral vein blood samples were collected with 5 ml lithium heparin tubes in the morning. Next, the blood samples were centrifuged at 13,000×*g* for 15 min at 4 °C. Finally, the obtained plasma samples were frozen at − 80 °C before metabolomics analyses.

### Plasma sample preparation

This study consists of two parts. In the first part of the study, two plasma preparation procedures were compared to each other regarding metabolome coverage, signal intensity and reproducibility. The most suitable plasma preparation procedure was then applied to an untargeted metabolomics analysis of the plasma samples of PAH patients and control group. The details and results of the first part of this study can be consulted in the Supplementary Materials. The selected plasma preparation protocol differs slightly from the conventional plasma preparation procedure. Instead of using only a mixture of cold organic solvents, this procedure also includes using proteinase K to relax the tertiary structure of the proteins in the plasma sample. This leads to a higher metabolome coverage, a higher signal intensity and better signal reproducibility. All details of the proteinase K plasma preparation procedure were previously described^16^.

To 50 µl of plasma, 5 µl of an internal standard solution (1 µg/ml solution of 1-(4-fluorobenzyl)-5-oxoproline in methanol) was added. Additionally, 1 µl of a 250 mM CaCl_2_-solution and 1 µl of a 20 mg/ml proteinase K-solution were added. Plasma samples were incubated in the Shaker-Incubator (SI-45, Hangzhou Allsheng Instruments, China) for 15 min at 37 °C. Then, 150 µl of a cold organic solvent was added to precipitate the proteins. The cold organic solvent was methanol:ethanol mixture (1:1 v/v), which was stored at − 80 °C before use. The sample was vortexed for 5 min and stored at − 20 °C for 60 min, followed by centrifugation at 13,000×*g*, at 4 °C for 15 min. The obtained supernatant was filtered by a titan syringe filter and then transferred into an amber glass HPLC vial with a 200 μl insert.

**Figure 1:**
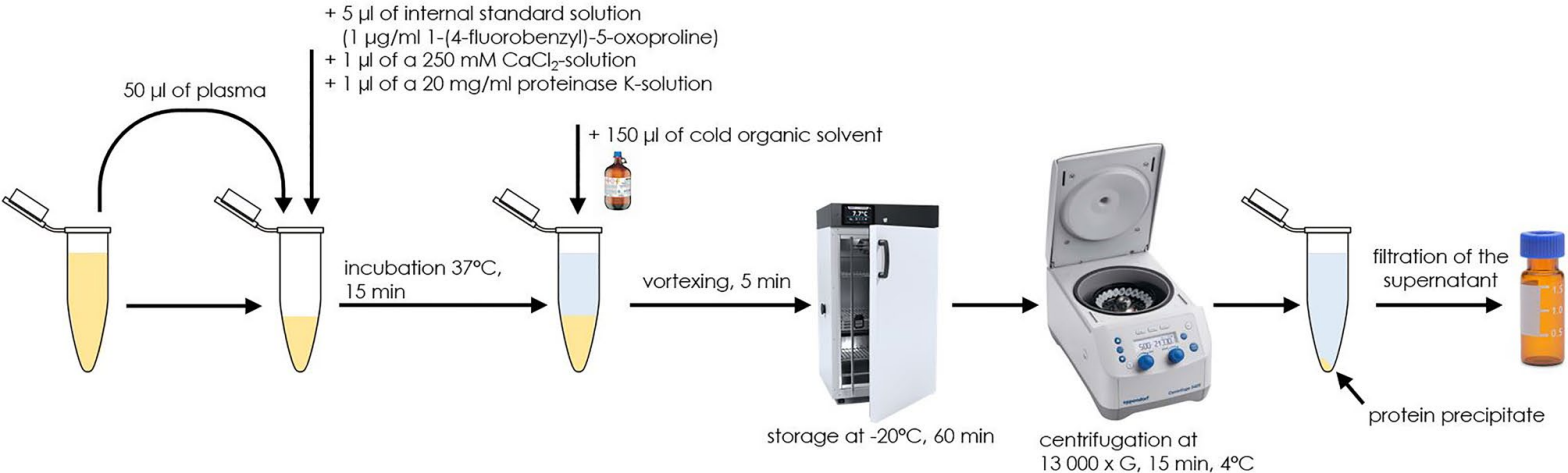
A detailed overview of the plasma preparation procedure that was used to prepare the plasma samples of PAH patients and control group for the analysis with untargeted metabolomics.

For the preparation of the quality control (QC) samples, a pooled plasma sample was prepared by collecting 20 µl of each plasma sample in the same vial. Next, the vial was vortexed to homogenize the pooled plasma sample. After this step, the preparation of the QC samples was analogous to the plasma preparation protocol, as described in Fig. 1.

### Analytical measurements

The HPLC analysis was performed on an Agilent Technologies 1200 series HPLC system, using a gradient elution on a Zorbax Extend-C18 column (2.1 × 100 mm, 3.5 µm, Agilent Technologies, Waldbronn, Germany). The plasma metabolomic fingerprints were measured on a TOF mass analyzer from the Agilent Technologies 6224 series, equipped with a dual ESI source. The samples were analyzed in a randomized order, with one QC sample after every eight samples. All chromatographic runs were first completed in positive ionization mode, followed by negative ionization mode. All chromatographic and mass spectrometer parameters are described in detail in the Supplementary Materials (Fig. S2).

### Data processing, statistical analyses and metabolite identification

After analytical measurements, the acquired raw data were processed using the Agilent MassHunter Profinder 10.0 software (Agilent Technologies, Waldbronn, Germany). A detailed overview of the settings used during the molecular feature extraction is included in the Supplementary Materials (Table S1). Subsequently, the data were filtered in RStudio (Posit Software, Boston, MA, United States), and the signals were normalized based on the intensity of the internal standard. The parameters for the data filtration are described in the Supplementary Materials. Next, the normalized signals were corrected with a support vector machine for regression (SVR) script in Matlab R2016b (Mathworks, Natrick, MA, United States). This algorithm corrects the variations in the sample signals based on the order of analysis of the QC samples. The multivariate statistical analyses were performed in SIMCA 16 (Sartorius Stedim Biotech, Umeå, Sweden). First, a principal component analysis (PCA) was applied to verify if the QCs were clustered together to confirm the reproducibility of the applied methods and to evaluate potential outliers. Additionally, an orthogonal partial least squares discriminant analysis (OPLS-DA) plot was generated to identify the variables of importance on the projection (VIPs > 1.0) and |*p(corr)*|> 0.2, which had the highest predictive capacity to distinguish the PAH patients from the control group. The obtained OPLS-DA models were k-fold cross-validated and the R2, Q2 and *p*_*CV-ANOVA*_ values were evaluated.

CEU MassMediator (http://ceumass.eps.uspceu.es/mediator) was used to annotate the statistically significant features based on their monoisotopic mass and isotopic distribution pattern. The identification was then confirmed by an MS/MS fragmentation pattern analysis. To obtain the MS/MS spectra, one QC sample was injected in both positive and negative ionization modes and analyzed via the automated Q-TOF iterative MS/MS acquisition mode. This acquisition mode allows for the acquisition of several different MS/MS spectra during the five consecutive runs. In addition, precursors that were selected for MS/MS fragmentation during previous runs are automatically excluded from the fragmentation so that the less abundant ions can be fragmented during the later runs. The settings and instrument parameters for the fragmentation pattern analysis are described in more detail in the Supplementary Materials.

## Results

### Comparison of plasma preparation procedures

**Figure 2:**
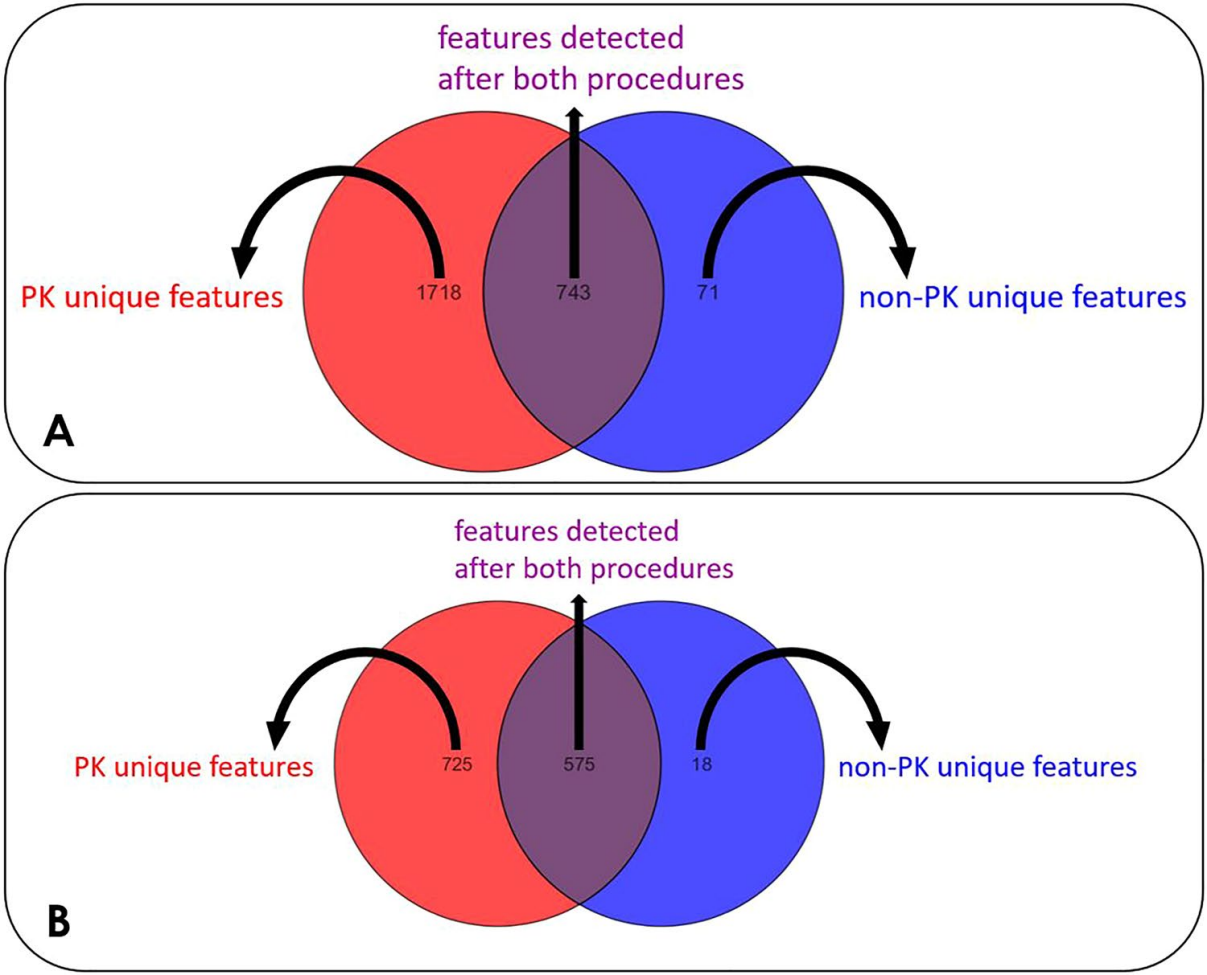
The number of features that were found after applying the proteinase K (PK) sample preparation procedure is depicted in the red spheres, in both positive (**A**) and negative (**B**) ionization modes. The blue spheres represent the number of features that were found after the application of the conventional plasma preparation procedure (non-PK). The features that were found with both procedures are depicted in the overlapping area.

3098 features were extracted from the raw data in positive ionization mode, whereas in negative ionization mode, 1532 features were obtained. After the filtration steps, 2532 features were left for positive and 1318 for negative ionization mode, respectively. Figure 2 shows the number of features that were observed after each plasma preparation procedure in both positive (Fig. 2A) and negative ionization (Fig. 2B) modes. In both ionization modes, the number of features is considerably higher after applying the proteinase K (PK) plasma preparation procedure compared to the conventional plasma preparation procedure (non-PK). In positive ionization mode, 1718 additional features were detected after the PK procedure, compared to only 71 additional features after applying the non-PK plasma preparation procedure. A similar trend can be observed for the negative ionization mode: 725 other features were detected after using the PK procedure, whereas only 18 additional features were detected after the non-PK procedure. Thus, the proteinase K plasma preparation procedure increased the metabolome coverage considerably for both ionization modes.

The vast amount of features detected with both procedures have a considerably higher intensity after applying the PK procedure. In positive ionization mode, 743 features were found after both plasma preparation procedures, however, 734 had a higher intensity after the proteinase K procedure. The negative ionization data set results are analogous: 499 out of 575 features had a higher abundance after the proteinase K procedure. The fold changes in the intensities of the features in the PK procedure over the non-PK procedure are illustrated in Fig. S3 in the Supplementary Materials. In positive ionization mode, most features had a 3 to fourfold higher intensity after the application of the PK procedure, compared to the non-PK procedure (see Fig. S3A). For negative ionization mode, most features had a fold change of 1 to 2 times higher after the PK procedure (see Fig. S3B). Hence, the vast majority of features detected after both plasma preparation procedures had a higher intensity after the proteinase K plasma preparation procedure. This implies that the proteinase K plasma preparation procedure increases the intensity of most features in both positive and negative ionization modes.

Not only the metabolite coverage and the metabolite intensity increase after applying the proteinase K procedure, but the measurements are also more reproducible after the PK procedure. The comparison of the coefficients of variation of the features can be found in Fig. S4A in the Supplementary Materials. In positive ionization mode, a larger proportion of the features had a coefficient of variation under 5% after the proteinase K procedure, compared to the conventional plasma preparation procedure. The relative proportion of features with a coefficient below 20% for the negative ionization data set also increased after applying the proteinase K procedure. The relative increase in the number of features with a lower coefficient of variation suggests that the proteinase K procedure enables more reproducible measurements than the conventional plasma preparation procedure. Figure S4B (in the Supplementary Materials) shows the PCA plots for 10 pooled QC samples which were prepared with the PK and conventional procedures, in both positive (panel A) and negative (panel B) ionization modes.

**Figure 3:**
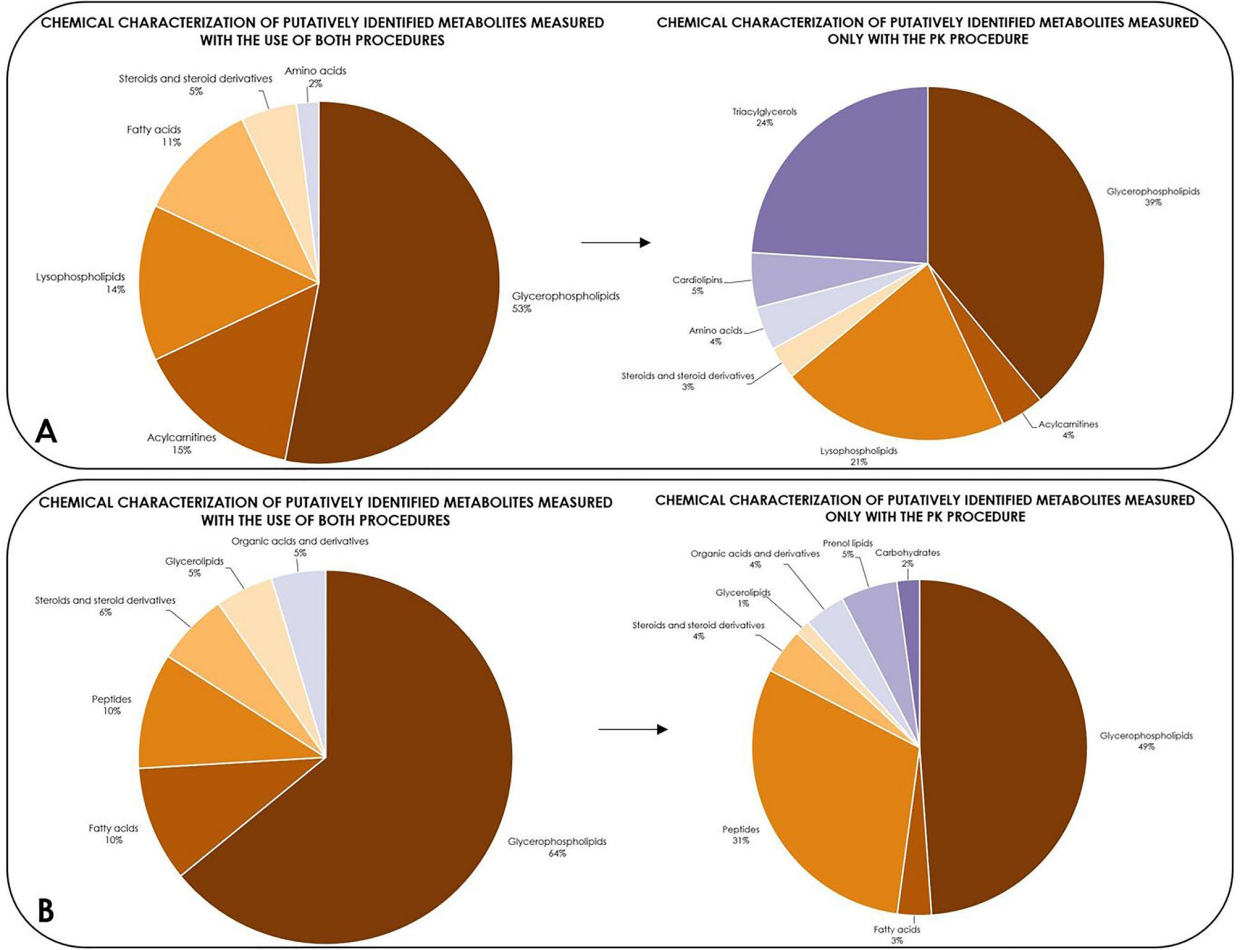
Changes in the chemical characterization between the annotated metabolites that were measured with both plasma preparation procedures (left) and those unique to the proteinase K procedure (right) in both positive (**A**) and negative (**B**) ionization modes.

After the putative annotation of the metabolites found for both procedures, their chemical groups were visualized in Fig. 3. After applying the proteinase K procedure, a larger proportion of the metabolites in the positive ionization data set were characterized as lysophospholipids and cardiolipins, which are lipophilic compounds. For the negative ionization data set, there was an increase in both lipophilic and more hydrophilic compounds because significant increases were observed in the prenol lipid and peptide groups. It should be noted that the increase in peptides is a direct result of the enzymatic action of proteinase K. However, the tripeptides make up less than one-third of the total metabolites. Considering that the total number of features increased from 593 to 1300 after the proteinase K procedure, there is still a net gain of approximately 300 additional features after excluding the peptides.

### Metabolomic signature of PAH patients

After the preprocessing of the raw data, 2498 features were exported from positive ionization mode, whereas in negative ionization mode, 1574 features were obtained. After the data filtration, 1986 features remained for the positive ionization mode, whereas in negative ionization mode, 1298 features were selected. The PCA model for the positive ionization mode data set was built on UV-scaled and log-transformed data. For the negative ionization data set Pareto scaling was used in combination with log transformation. The scores plot for the PCA model of the positive ionization data set can be found in panel A of Fig. S5 in the Supplementary Materials, along with the scores plot of the PCA model of the negative ionization data set, which was included in panel B of the same figure. The PCA models were developed to verify the stability of the analytical system based on the clustering of the QC samples. For both ionization modes, the QC samples are clustered together, but the PAH patients and control group are distributed over a far greater area. This indicates that the analytical variability between the samples will be negligible in comparison to their biological variability. After verifying the analytical stability, an OPLS-DA model was built for the data sets of both ionization modes. For both ionization modes UV scaling was used in combination with log transformation to build the OPLS-DA models. The scores plots for the OPLS-DA models, including k-fold cross-validation parameters (R2, Q2 and *p*_*CV-ANOVA*_) can be found in Fig. S6. The OPLS-DA models were built to select the important variables to distinguish the PAH patients from the control group. The variables with a VIP score higher than 1.0 and |*p(corr*)| higher than 0.2 were selected for further statistical analysis.

The chemical and statistical characteristics of the identified metabolites were presented in Tables 2 and 3.

**Table 2:** Chemical characteristics of identified statistically significant metabolites in plasma of PAH patients as compared to a control group.

Experimental mass	RT (min)	ESI mode	Fragmentation pattern (m/z values of fragments)	Metabolite
188.1158	4.6	+	189.0840, 171.1150, 143.1187, 132.1010, 86.0966,	Acetyl-lysine
341.2564	6.1	+	342.2643,283.1909, 181.1607, 144.1019, 85.0283	Dodecenoylcarnitine
399.3373	8.8	+	400.3430, 341.2707, 239.2679, 85.0285	Palmitoylcarnitine
525.2871	11.5	+	526.2976, 472.1580, 385.2735, 243.1731, 62.0601	LPE(22:6)
543.3409	9.9	+	544.3389, 485.2641, 184.0735, 104.1069, 86.0964	LPC(20:4)
584.2624	14.0	+	ESI+: 585.2710, 299.1391, 287.1392 ESI−:583.2554, 285.1246	Bilirubin
719.5816	17.5	+	720.5907,537.9430,413.3129,319.1944,184.0733,86.0970	PC(32:0)
728.5826	16.2	+	729.5917,652.5106,283.3260,184.0736,86.0961	SM(36:2)
831.5751	16.3	+	832.5896, 550.3380,184.0734, 124.9994, 86.0976	PC(40:7)
859.6019	15.2	+	860.6147, 801.5405, 677.5516, 184.0708, 146.9808, 86.0964	PC(42:7)
108.0574	5.3	−	106.0422, 105.0339, 79.5673	o/m/p cresol
204.0897	2.7	−	159.0934, 155.0834	Tryptophan
408.2857	8.8	−	392.3395, 363.1245, 346.2586	Trihydroxycholanoic acid
426.3693	14.2	−	407.3537, 381.3610	Hexacosanedioic acid
477.2845	11.2	−	476.2773, 279.2330, 196.0401, 140.9949	LPE(18:2)
481.3162	11.9	−	480.3096, 283.2649,152.9970,140.9948	LPE(18:0)
501.2844	11.2	−	500.2772, 457.2291, 303.2319,140.9948, 78.9591	LPE(20:4)
507.3321	12.2	−	506.3249, 463.2829, 309.2699, 291.2689, 140.0115	LPE (20:1)
509.3483	13.1	−	508.3411, 311.2945, 140.9958, 78.9586	LPE (20:0)
539.3212	10.9	−	538.3140, 451.2832, 297.2796, 152.9958, 78.9591	LPS(19:0)
727.5506	16.1	−	464.3143, 446.3035, 281.2468, 279.2329,140.0121	PE(18:0/18:2)

*RT* retention time, *ESI* electrospray ionization, *LPE* lysophosphatidylethanolamine, *LPC* lysophosphatidylcholine, *PC* phosphatidylcholine, *SM* sphingomyelin, *LPS* lysophosphatidylserine.

**Table 3:** Statistical characteristics of the identified metabolites significantly changed in the plasma samples of PAH patients.

Metabolite	*|p(corr)|*	VIP	% change (PAH *vs* Control)
Acetyl-lysine	0.33	1.3	− 79
Dodecenoylcarnitine	0.49	1.9	− 73
Palmitoylcarnitine	0.43	2.3	− 72
LPE(22:6)	0.42	2.1	− 71
LPC(20:4)	0.36	1.4	− 83
Bilirubin	0.38	2.1	39
PC(32:0)	0.41	1.4	− 65
SM(36:2)	0.4	1.7	28
PC(40:7)	0.4	1.6	− 81
PC(42:7)	0.41	1.3	− 87
o/m/p cresol	0.28	1.3	− 76
Tryptophan	0.25	1.3	− 74
Trihydroxycholanoic acid	0.28	1.1	− 97
Hexacosanedioic acid	0.49	2.1	− 98
LPE(18:2)	0.21	1.1	− 94
LPE(18:0)	0.26	1.4	− 81
LPE(20:4)	0.20	2.0	− 93
LPE(20:1)	0.27	1.4	− 92
LPE(20:0)	0.27	1.3	− 92
LPS(19:0)	0.22	1.3	− 31
PE(18:0/18:2)	0.21	1.2	− 84

## Discussion

The evaluation of metabolic features and biochemical pathways associated with PAH can provide a holistic insight into the molecular mechanisms, improve the current diagnostic or treatment schedules, identify subphenotypes of the disease and propose new therapeutic targets. The results of recently published studies have confirmed that metabolic dysregulation is a common feature of various clinical types of PAH^8,11,12,17–20^. Herein, a global plasma untargeted metabolomics with the use of the LC–MS technique was applied to characterize the metabolic phenotype of PAH patients. Moreover, a sample preparation procedure with the use of PK was used. PK is a well-known proteolytic enzyme that belongs to the serine protease class produced by the *Tritirachium album*^21^. PK has a broad cleavage specificity and breaks the peptide bond adjacent to the carboxylic group of aliphatic and aromatic amino acids, which is a helpful characteristic for protein digestion in biological matrices^22^. Furthermore, plasma provides a hydrophilic environment and limits the solubility of hydrophobic metabolites such as fatty acids, lipids, steroids and thyroid hormones. The efficient transport and distribution of these metabolites in plasma in vivo are provided by their interaction with proteins, mainly albumin^15^. Hence, the immediate plasma protein precipitation, standard for the plasma untargeted metabolomics workflow, leads to co-precipitation of a significant fraction of mainly hydrophobic metabolites, which can be a limiting factor for disease related metabolomic analyses. Therefore, an additional step, namely the digestion with PK to release strongly associated metabolites, was included in the sample preparation procedure before the plasma protein precipitation. To the best of the authors’ knowledge, this approach was applied for the first time in the case of PAH.

In this study, the differentially altered metabolites in the disease group were mainly related to the lipid metabolism (Tables 2 and 3). The lipid metabolic pathways include the synthesis of structural and functional lipids (such as phospholipids, glycolipids, sphingolipids, cholesterol, prostaglandins, etc.), which are characteristic of each individual tissue. The detailed description of lipid metabolism was previously summarized in terms of their synthesis and degradation^23^. Lipids play a crucial role in lung physiology and pathophysiology. Besides their function as components of the biological membranes and as a pulmonary surfactant, phospholipids constitute a diverse group of intracellular signaling biomolecules, which are involved mainly in cell proliferation and apoptosis^24^. There are four major classes of phospholipids in thehuman plasma, namely phosphatidylethanolamines (PEs), phosphatidylserines (PSs), phosphatidylcholines (PCs), and sphingomyelins (SMs). In this study, a decrease in plasma PCs [PC(32:0), SM(36:2), PC(40:7), PC(42:7)], PE(18:0/18:2), lysophosphatidylethanolamines (LPEs) [LPE (22:6), LPE(18:2), LPE (18:0), LPE(20:4), LPE (20:1), LPE (20:0)] LPC(20:4) and lysophosphatidylserine (LPS 19:0) was found in PAH patients in comparison to the control group. The decreased levels of various phospholipids were observed in the plasma of chronic thromboembolic pulmonary hypertension patients (CTEPH)^17^, in bronchoalveolar lavage fluid of patients 10 days after pulmonary embolism^25^, and in a pulmonary embolism model in pigs^26^. Phospholipids are also sources of multiple cellular signalling molecules, including prostacyclin, which is known to be reduced in pulmonary hypertension^27^. Additionally, numerous sphingomyelins and phosphatidylcholines were significantly reduced in plasma of patients with PAH, relating to increased mortality^18^. SMs are the most abundant subclass of sphingolipids, among other subclasses, such as sphingosine, ceramides, and glycophospholipids. Low plasma levels of several SMs were associated with the disease severity in patients with the chronic obstructive pulmonary disease^28^. These lipid species, which are produced from phosphatidylcholines and ceramides by sphingomyelin synthase, are involved in transmembrane signalling and are associated with mitochondrial dysfunction and reduced insulin release^29^. Additionally, SMs serve source of ceramide, which directly regulates cell proliferation, apoptosis, migration, and autophagy^30^. This study found an increase in plasma SM(36:2) in PAH patients compared to the control group.

**Figure 4:**
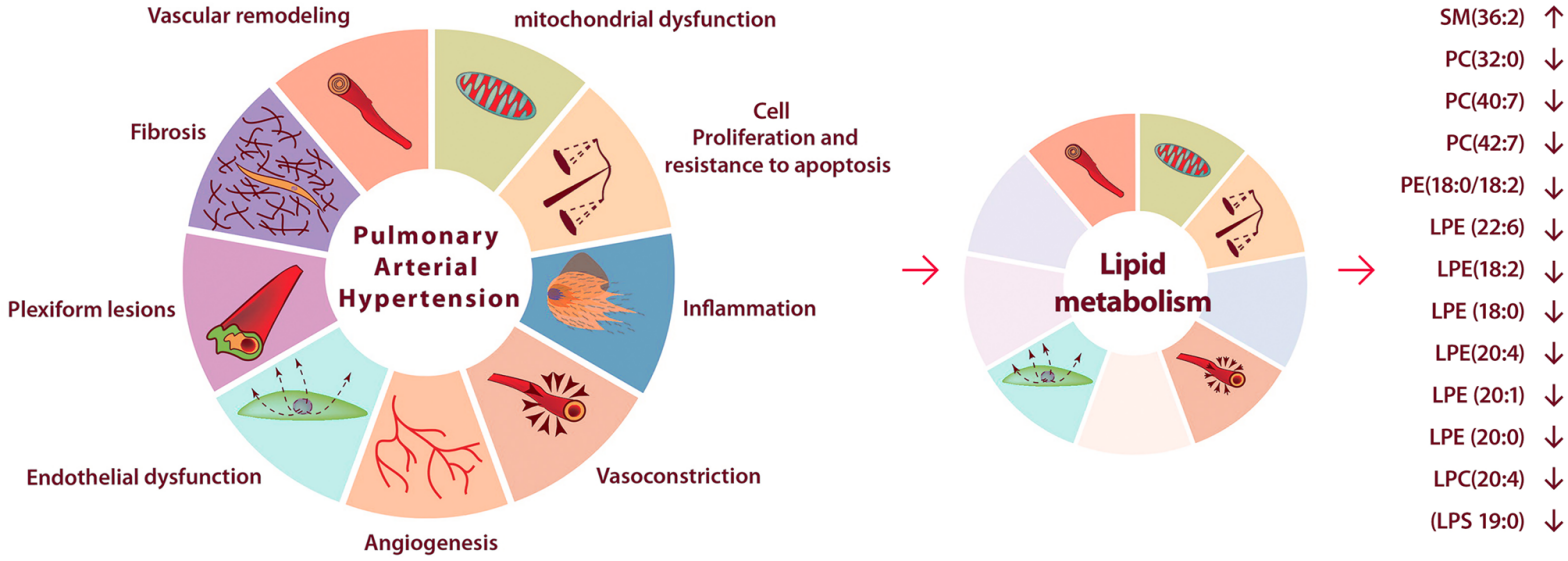
The general overview of potential relationships between the observed lipid alterations and molecular processes characteristic of the PAH pathobiology.

Besides the plasma phospholipid changes, our study found a decrease in hexacosanedioic acid, one of the dicarboxylic fatty acids, in PAH patients compared to the control group. Generally, dicarboxylic fatty acids result from the conversion of the terminal methyl group into a carboxyl group in the fatty acid moieties. The catabolism of fatty acids is typically generated by β-oxidation in the peroxisomes and/or mitochondria^31,32^. In the study of Zhao et al. a significant accumulation of dicarboxylic fatty acids in tissue samples derived from PAH patients was observed^33^. These results have suggested the increase of ω-oxidation in the smooth endoplasmic reticulum in addition to β-oxidation in the peroxisome or mitochondria of the PAH lung. This was additionally confirmed by gene array analysis, in which aldehyde dehydrogenase (ALDH18A1), a major enzyme in ω-oxidation, was significantly overexpressed in the PAH lung. Therefore, both the metabolomic and genetic changes have shown that ω-oxidation may become the major pathway for fatty acid oxidation if β-oxidation is insufficient to supply ATP as a crucial source of energy for the vascular remodeling process in PAH^33^. Taking together these results, the decrease of plasma hexacosanedioic acid in our study is probably caused by its accumulation in the lungs of PAH patients. The general overview of potential relationships between observed lipid alterations and molecular processes characteristic of PAH pathobiology was presented in Fig. 4.

This observational study has some limitations essential to notice. First, the findings presented here require verification in a larger population using targeted quantitative analyses to confirm the potential clinical relevance. Due to the rare frequency of PAH occurrence, the multicenter study, providing a decent number of samples, is required for the validation and selection of potential diagnostic markers of the disease. Frequently, the pathogenesis of PH can include features of different clinical groups, which can cause difficulties in recognition of the dominant etiology and application of suitable pharmacotherapy. Therefore, it should be underlined that metabolomics provides an essential molecular insight into the PAH pathogenesis, and each subtype of the disease is probably characterized by a specific metabolic signature.

## Conclusions

Plasma untargeted LC–MS based metabolomics with the proteinase K approach was applied to evaluate the metabolic changes associated with PAH. The main metabolic alterations in the PAH group were observed in the phospholipid metabolism. The PAH patients showed decreased levels of plasma PCs [PC(32:0), SM(36:2), PC(40:7), PC(42:7)], PE(18:0/18:2), LPEs [LPE(22:6), LPE(18:2), LPE (18:0), LPE(20:4), LPE (20:1), LPE (20:0)], LPC(20:4) and LPS(19:0) in comparison to the control group. Besides their function as components of the biological membranes, these metabolites are also involved in the intracellular signaling pathways, which are associated with cell proliferation and apoptosis. The results obtained during this study confirm the potential of (untargeted) metabolomics to identify the molecular characteristics of the pathophysiology of PAH.

The clinical relevance of this study constitutes the selection of a characteristic metabolic panel which could distinguish PAH patients from the control group. The selected metabolites have the potential to help in the early recognition of the disease. Moreover, RHC parameters are prognostic, however, the test is invasive, so the proposed metabolic indicators, detected in easily available and noninvasive plasma samples, could also be useful in the monitoring of the disease progression after the implementation of a specific pharmacological treatment.

### Supplementary Information

Supplementary Information.

## Data Availability

The datasets analyzed during the current study are available from the corresponding author on reasonable request.

## Abbreviations

6MWT: 6-Minute walk test
ALP: Alkaline phosphatase
ALT: Alanine aminotransferase
AST: Aspartate aminotransferase
BNP: Brain natriuretic peptide
CI: Cardiac index
CTEPH: Chronic thromboembolic pulmonary hypertension patients
DBP: Diastolic blood pressure
ECG: Electrocardiography
ESC: European Society of Cardiology
GGT: Gamma-glutamyltranspeptidase
HPAH: Heritable pulmonary arterial hypertension
HR: Heart rate
IPAH: Idiopathic pulmonary arterial hypertension
LPC(s): Lysophosphatidylcholine(s)
LPE(s): Lysophosphatidylethanolamine(s)
LPS(s): Lysophosphatidylserine(s)
mPAP: Mean pulmonary artery pressure
mRAP: Mean right atrial pressure
non-PK: Non-proteinase K
OPLS-DA: Orthogonal partial least squares discriminant analysis
PAH: Pulmonary arterial hypertension
PC(s): Phosphatidylcholine(s)
PCA: Principal component analysis
PCWP: Pulmonary capillary wedge pressure
PE(s): Phosphatidylethanolamine(s)
PH: Pulmonary hypertension
PK: Proteinase K
PL(s): Phospholipid(s)
PLT: Platelet count
PS(s): Phosphatidylserine(s)
PVR: Pulmonary vascular resistance
QC(s): Quality control sample(s)
RHC: Right heart catheterization
SBP: Systolic blood pressure
SM(s): Sphingomyelin(s)
SVR: Support vector machine for regression
VIP(s): Variable(s) importance in the projection
