# Supplementary material for: Plasma untargeted metabolomics with proteinase K discloses phospholipid signature associated with pulmonary arterial hypertension
Source: Sci Rep. 2023 Sep 15;13:15280. doi: 10.1038/s41598-023-42293-w (PMC10504264; doi:10.1038/s41598-023-42293-w)
Supplement: Supplementary file 1 — Supplementary Information. [file 41598_2023_42293_MOESM1_ESM.docx]

**Supplementary Materials**

**Materials and Methods**

- - Sample preparation

To compare both plasma preparation procedures to each other, a vial of pooled plasma was prepared by collecting 20 µl of each plasma sample in the same vial. The vial was vortexed to homogenize the sample. From this vial of pooled plasma, 10 samples were prepared with the conventional plasma preparation procedure and 10 with the proteinase K procedure, following a protocol that has been published earlier [1].


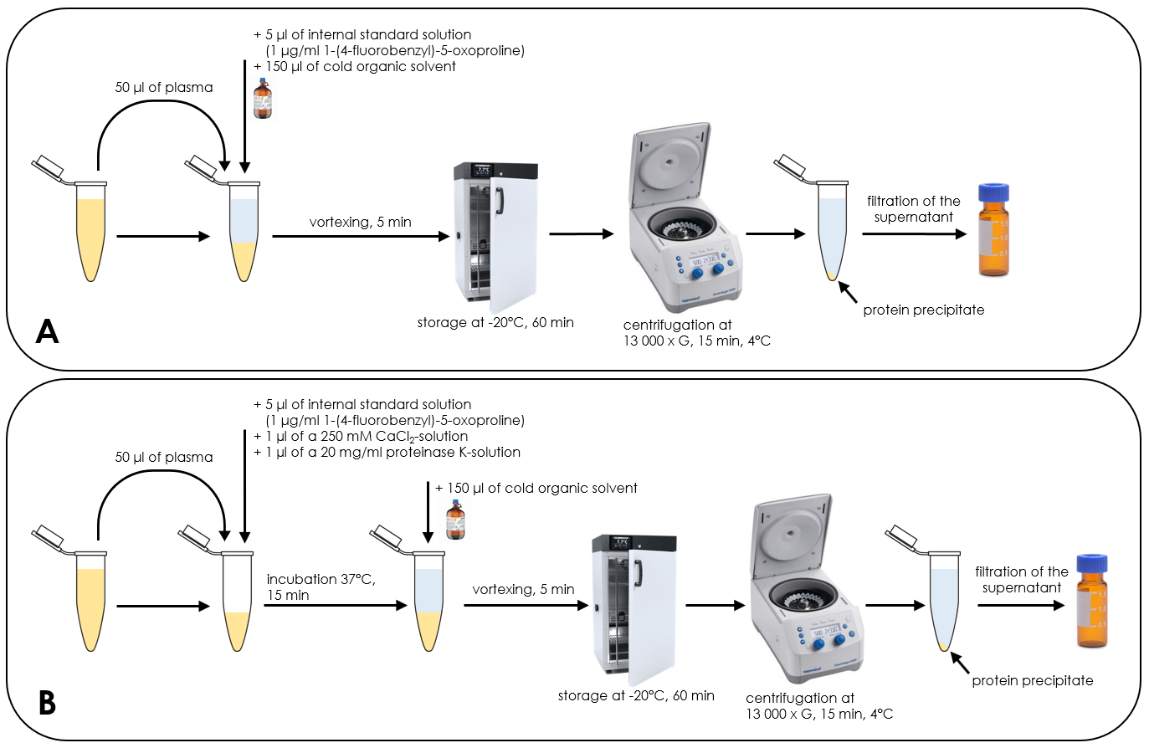
The conventional plasma preparation procedure is illustrated in part A of Figure S1. To 50 µl of pooled plasma, 5 µl of internal standard solution (1 µg/ml solution of 1-(4-fluorobenzyl)-5-oxoproline) was added. Subsequently, 150 µl of a cold organic solvent was added to precipitate the proteins. The cold organic solvent was a methanol:ethanol mixture (1:1 v/v), which was stored at -80°C before use. After the addition of the cold solvent, the sample was vortexed for five minutes and stored in -20°C for 60 minutes. Next, the samples were centrifugated at 4°C and 13000 x G for 15 minutes. Lastly, the supernatant was filtered by syringe nylon filters and transferred into an amber vial with a 200 μl glass insert.

Figure S1: An illustration of the detailed methodology of the two plasma preparation procedures that were evaluated and compared in terms of metabolome coverage, signal intensity and signal reproducibility. A: the conventional plasma preparation procedure, which uses cold organic solvents to precipitate the proteins in the sample. B: the plasma preparation approach employing additional incubation with proteinase K before protein precipitation and metabolite extraction with the use of various organic solvents.

The proteinase K procedure is illustrated in part B of Figure S1. To 50 µl of pooled plasma, 5 µl of internal standard solution (1 µg/ml solution of 1-(4-fluorobenzyl)-5-oxoproline) was added. Additionally, 1 µl of a 250 mM CaCl_2_-solution and 1 µl of a 20 mg/ml proteinase K-solution was added. Plasma samples were incubated in the Shaker-Incubator (SI-45, Hangzhou Allsheng Instruments, China) for 15 minutes at 37°C. 150 µl of a cold organic solvent was added to precipitate the proteins. The cold organic solvent was a methanol:ethanol mixture (1:1 v/v), which was stored at -80°C before use. The sample was vortexed for five minutes and stored in -20°C for 60 minutes, followed by centrifugation at 13000 x G at 4°C for 15 minutes. The obtained supernatant was filtered by a nylon syringe filter and transferred into an amber glass HPLC vials with a 200 μl insert.

- - Analytical measurements

The HPLC analysis was performed using a Zorbax Extend-C18 column (2,1 × 100 mm, 3,5 micron, Agilent Technologies (Waldbronn, Germany)) on an Agilent Technologies 1200 series HPLC system. The column temperature was set to 35°C.


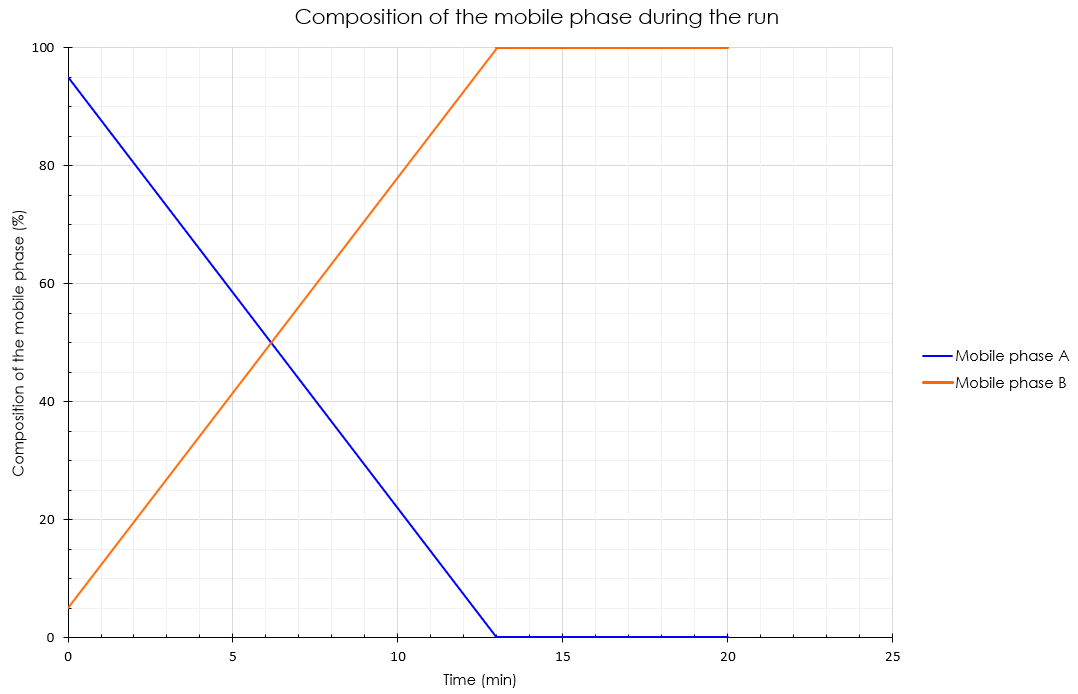
A gradient elution of mobile phase was applied at the continuous flow rate of 0,5 ml/min. Mobile phase A was 0,1% formic acid in deionized water and mobile phase B was 0,1% formic acid in methanol. From 0 to 13 min, mobile phase B eluted from 5% to 100% and was then kept for 7 min at 100% of B. An equilibration time of 5 min was used between the runs. The composition of the mobile phase during the runs is illustrated in Figure S2.

The injection volume was 2 μl per sample. The plasma metabolomic fingerprints were measured with a TOF mass analyzer from Agilent Technologies 6224 series, equipped with a dual ESI source. The scan rate was 1,51 spectra/s in the m/z-range 50 to 1000.

Figure S2: Illustration of the composition of the mobile phase during the runs. Mobile phase A was 0,1% formic acid in deionized water and mobile phase B was 0,1% formic acid in methanol.

During sample analysis a reference solution with four reference masses is dispensed continuously through the dual ESI to provide accurate mass measurements. The capillary and fragmentor voltage were 3250 V and 150 V, respectively. The skimmer voltage was set to 65 V. The flow rate and pressure of the nebulizer gas was 10 l/min and 45 psig, respectively. The ion source temperature was set to 350°C.

The samples were analyzed in a randomized order with one QC sample every eight samples. All chromatographic runs were completed first in positive ionization mode, followed by negative ionization mode.

The fragmentation pattern analysis was performed on an Agilent Technologies 1290 Infinity II series HPLC system, using a gradient elution on a Zorbax Extend-C18 column (2,1 × 100 mm, 3,5 micron, Agilent Technologies (Waldbronn, Germany)). The column temperature was set to 35°C and the injection volume was 5 μl per sample. The composition of the mobile phase during the fragmentation pattern analysis was identical to the protocol that was earlier described in the analytical measurements section of part I (see also Figure S2).

The MS/MS spectra were recorded on a Q-TOF-MS analyzer from the Agilent Technologies 6546 series, equipped with a dual ESI source. The MS scan rate was 1.1 spectra/s in the m/z-range 61 to 1700. For MS/MS, the scan rate was slightly faster at 1.01 spectra/s, however the m/z-range stayed the same.

During the sample analysis a reference solution with four reference masses was dispensed continuously through the dual ESI to ensure accurate mass measurements. The capillary and fragmentor voltage were 3250 V and 150 V, respectively. The skimmer voltage was set to 65 V. The flow rate and pressure of the sheath gas was 11 l/min and 45 psi, respectively. The ion source temperature was set to 350°C.

To obtain the MS/MS spectra, one QC sample was injected five consecutive times in both positive and negative ionization modes and analyzed via the automated Q-TOF iterative MS/MS acquisition mode. This acquisition mode enables the user to record different MS/MS spectra during each consecutive run. Precursors that were selected for MS/MS fragmentation during previous runs are automatically excluded for the fragmentation, so that the less abundant ions can be fragmented during the later runs.

| Step 1: Extraction parameters for the molecular feature extraction | |
| --- | --- |
| Time (min) | 0 - 20 |
| Cut-off value for noise | Minimal 300 counts |
| Ion species allowed | H+, Na+, K+ and loss of H2O |
| Step 2: Compound filters | |
| Mass filter (based on ion count) | Include all features |
| Mass defects (indeterminate masses) | Include features |
| Step 3: Compound binning and alignment | |
| Retention time tolerance | 0,1% + 0,15 minutes |
| Mass tolerance | 20 ppm + 2 mDa |
| Step 4: Post-processing filters | |
| Absolute peak height for features | Minimal 1000 counts |
| Score (MFE) | Minimal 70 |
| Minimum filter match | The compound must be present in at least 50% of the files in at least 1 sample group |
| Step 5: Find by ion - matching tolerances and scoring | |
| Chromatogram extraction mass tolerance | ± 35 ppm |
| Chromatogram extraction retention time | 1,5 minutes |
| Match tolerance for mass | ± 30 ppm |
| Match tolerance for retention time | 0,15 minutes |
| Step 6: Find by ion - peak integration and filtering | |
| Filter type | Peak height |
| Absolute height | 1000 counts |
| Step 7: Find by ion - spectrum extraction | |
| All default parameters | |
| Step 8: Find by ion - post processing filters | |
| Score | Minimal 60 |
| Minimum filter match | The compound must be present in at least 50% of the files in at least 1 sample group |

*Table S1: Detailed overview of the settings for the recursive feature extraction in the Agilent MassHunter Profinder 10.0 software.*

- - Data processing

After the chromatographic analysis, the acquired raw data was processed using the Agilent MassHunter Profinder 10.0 analysis software (Agilent Technologies, Waldbronn, Germany). A detailed overview of the settings used during the molecular feature extraction is included in Table S1.

Subsequently, the data was filtered in Mass Profiler Professional B.02.01 (Agilent Technologies, Waldbronn, Germany). The features had to be absent in 100% of the blanks and present in 80% of the samples in one group to be retained after the filtration. The data matrix was separated in three parts: features that were measured uniquely after the proteinase K sample preparation procedure, features that were measured uniquely after the conventional plasma preparation procedure and lastly the features that were measured after both plasma preparation procedures. After the filtration, all signals were normalized based on the internal standard.

**Results**


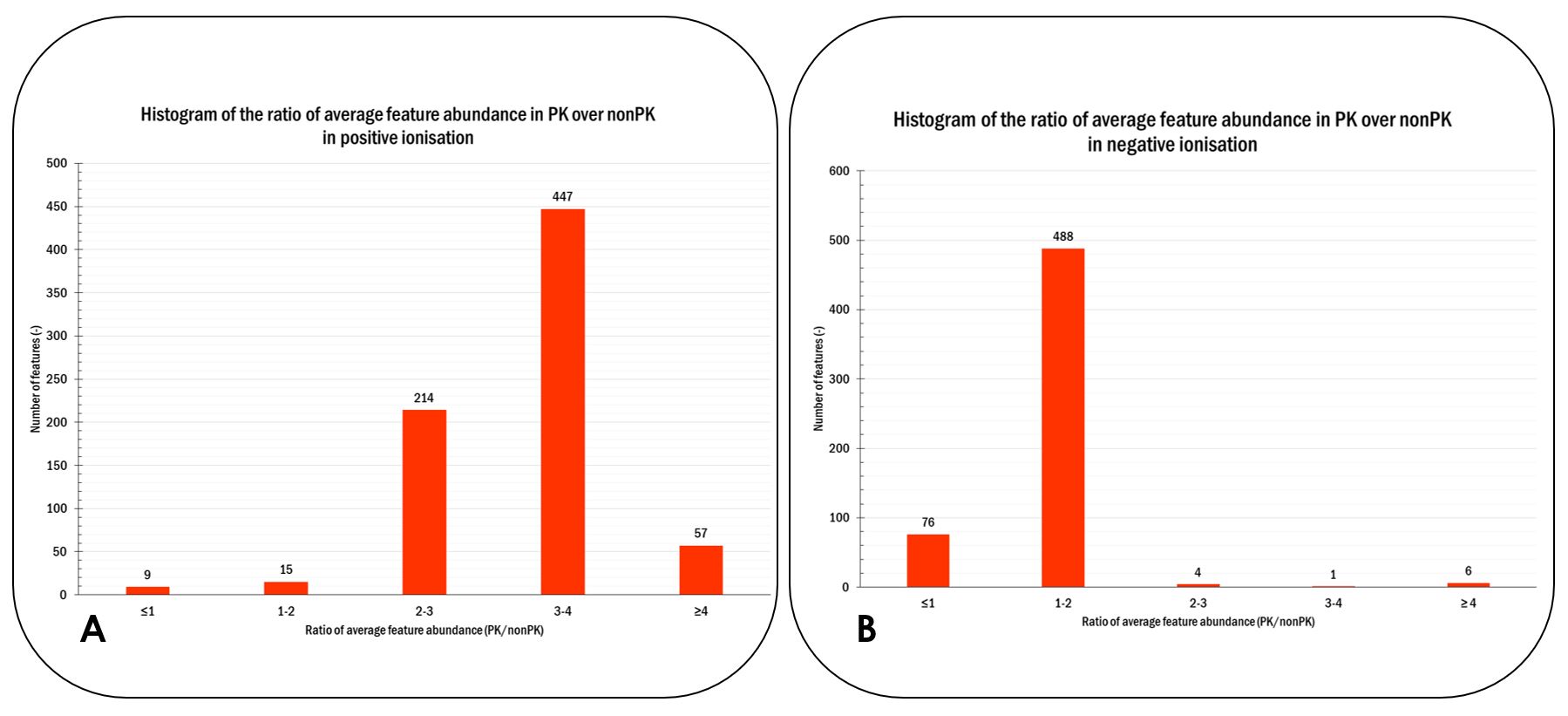


Figure S3: Histograms of the fold changes (PK over non-PK) of the features that were detected after both the PK and non-PK plasma preparation procedures in positive (A) and negative (B) ionization mode.


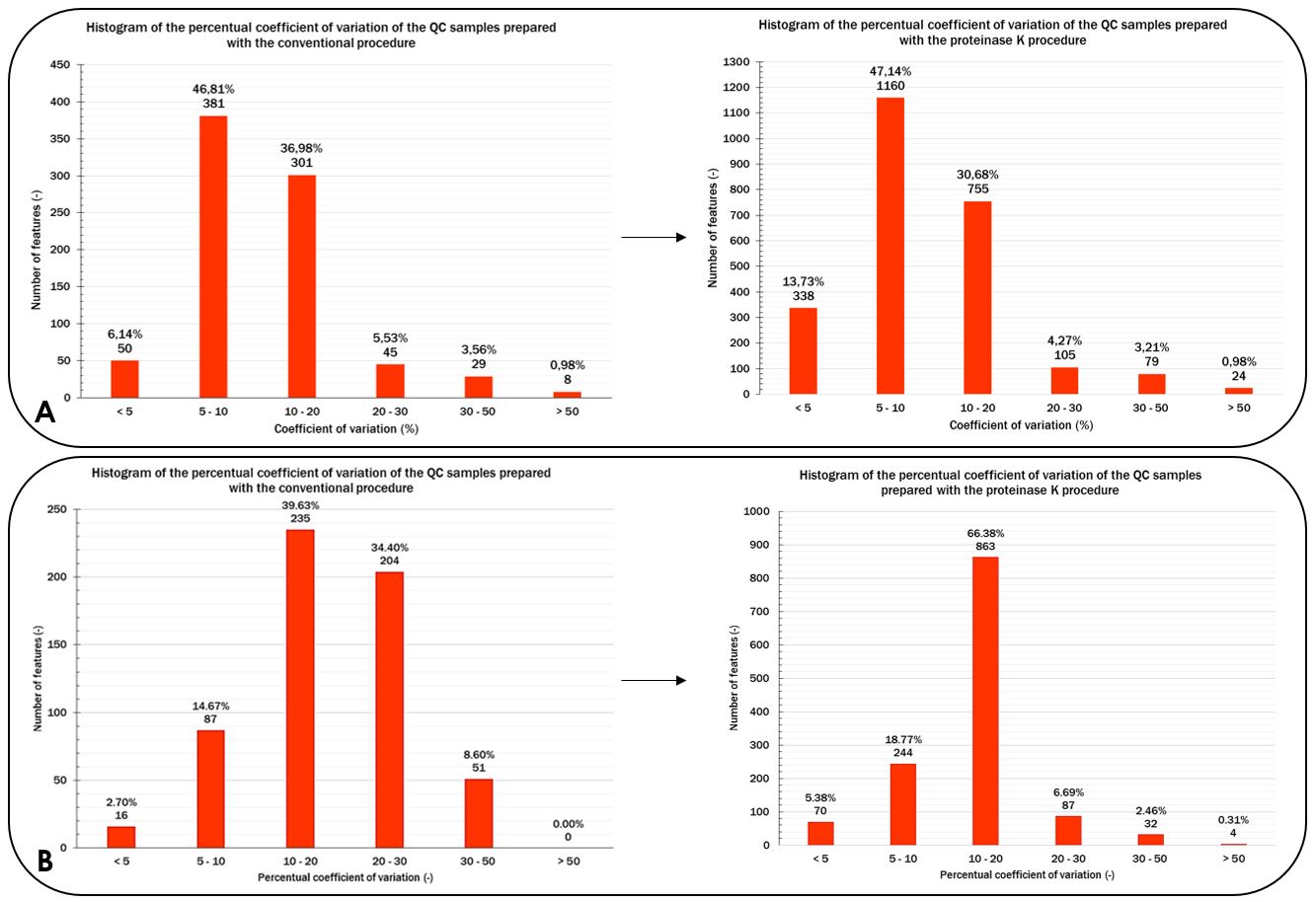


Figure S4A: A comparison of the percentual coefficient of variation after the conventional (left side) and proteinase K (right side) plasma preparation procedures in both positive (A) and negative (B) ionization modes.


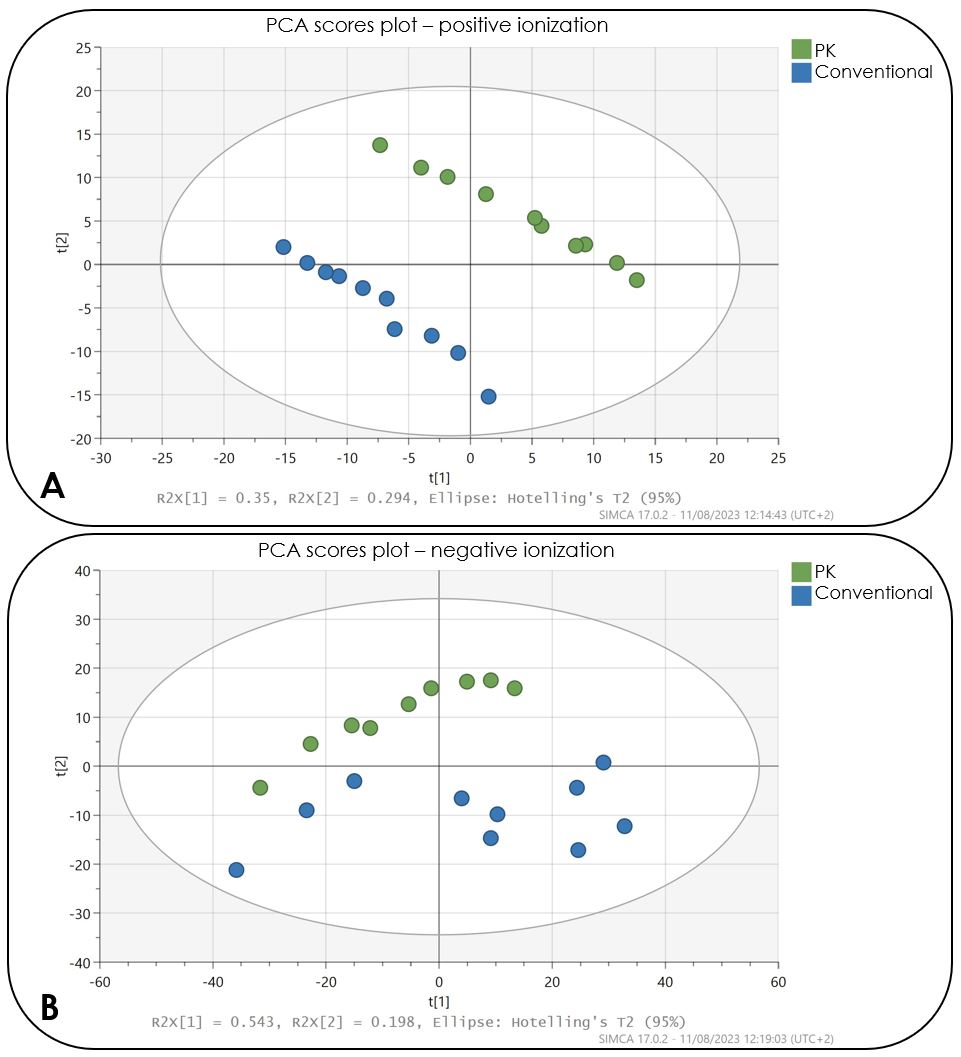


Figure S4B. PCA plots for 10 pooled QC samples prepared with PK procedure (green circles) and conventional procedure (blue circles) in both positive (panel A) and negative (panel B) ionization modes.


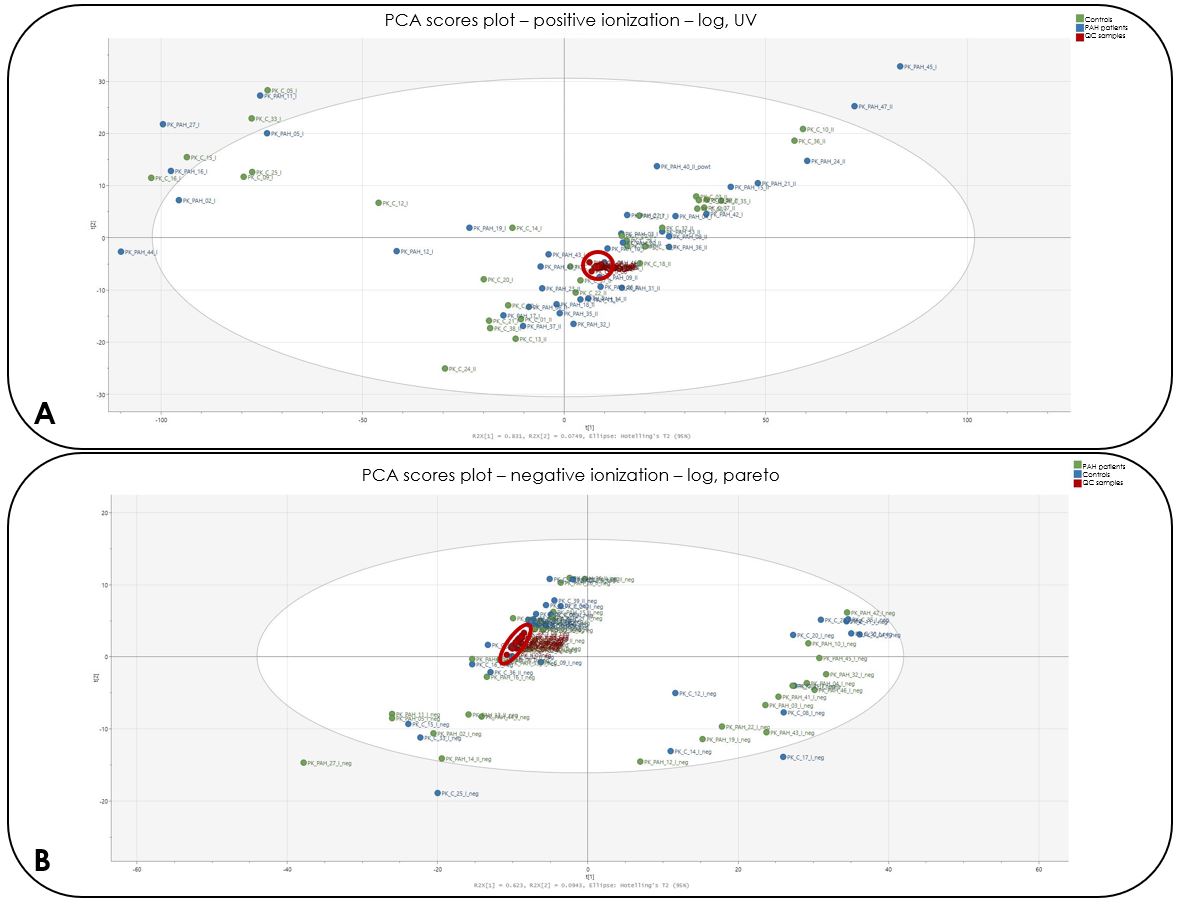


Figure S5: the PCA scores plots for positive (panel A) and negative (panel B) ionization mode for all three groups of samples (PAH patients, controls, and QC samples). The PCA model of positive ionization mode was built after UV scaling and log transformation of the data. For negative ionization mode, pareto scaling and log transformation was performed. The group colors are indicated in the legend on the top right corner of each scores plot.


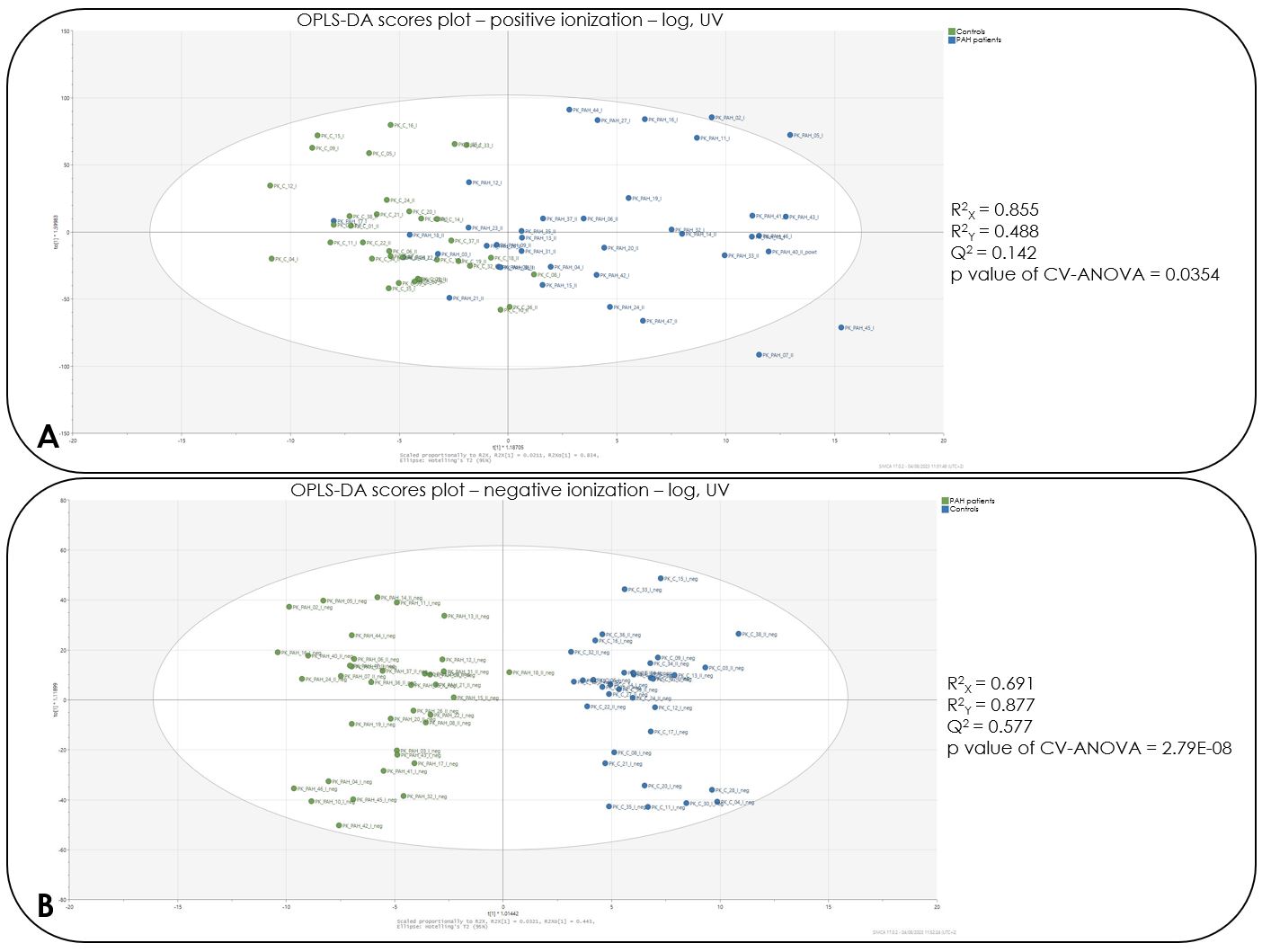


Figure S6: OPLS-DA scores plots for the positive (panel A) and negative (panel B) ionization modes for PAH patients and healthy controls. The OPLS-DA models of both positive and negative ionization mode were built after UV scaling and log transformation of the data. The group colors are indicated in the legend on the top right corner of each scores plot.

**References**

[1] Wawrzyniak R, Kosnowska A, Macioszek S, Bartoszewski R, Markuszewski MJ: New plasma preparation approach to enrich metabolome coverage in untargeted metabolomics: plasma protein bound hydrophobic metabolite release with proteinase K. *Sci Rep* 2018, 8:1. DOI: 10.1038/s41598-018-27983-0
